# Comparative Efficacy of Different Therapeutic Interventions in Eustachian Tube Dysfunctions: A Cross-Sectional Analysis

**DOI:** 10.3390/diagnostics14121229

**Published:** 2024-06-12

**Authors:** Sarah Alshehri, Abdullah Musleh

**Affiliations:** 1 Otology and Neurotology, Department of Surgery, College of Medicine, King Khalid University, Abha 61423, Saudi Arabia; 2 Otolaryngology, Head and Neck Surgery Department of Surgery, College of Medicine, King Khalid University, Abha 61423, Saudi Arabia; amusleh@kku.edu.sa

**Keywords:** Eustachian tube dysfunction, ETDQ-7, audiometry, tympanometry, nasopharyngoscopy

## Abstract

Eustachian tube dysfunction (ETD) affects a significant portion of the population, manifesting symptoms that impact the quality of life. Despite the prevalence of ETD, there remains a notable gap in comprehensive studies exploring the condition’s dynamics within specific demographic contexts, particularly within Saudi Arabia. This study aimed to assess the prevalence and severity of ETD across different demographic groups, to evaluate the efficacy of various treatment modalities, and to identify key predictors of treatment response in a Saudi Arabian cohort. A cross-sectional study was conducted from June 2022 to May 2023 in tertiary care hospitals in the Aseer region, Saudi Arabia. Participants included adults diagnosed with ETD, assessed through clinical symptoms, otoscopic examinations, audiometric evaluations, tympanometry, and the ETDQ-7 questionnaire. The study incorporated advanced diagnostics such as nasopharyngoscopy and pressure equalization tube function tests and involved 154 participants, revealing significant variations in ETD severity, with the 46–60 age group exhibiting the highest mean ETDQ-7 score of 4.85, and urban residents displaying lower severity scores compared to rural counterparts. Pharmacological interventions were most effective, achieving the highest symptom relief and audiological improvement rates of 87.78%. Multivariate regression highlighted age, geographic location, and treatment modality as key predictors of treatment efficacy, with notable interaction effects between climate conditions and treatment types influencing outcomes. The findings underscore the heterogeneity in ETD presentation and the differential efficacy of treatment modalities.

## 1. Introduction

Eustachian tube dysfunction (ETD) is a prevalent condition affecting a significant proportion of the population at some point in their lives [1]. It involves the malfunctioning of the Eustachian tube, which connects the middle ear to the nasopharynx and plays a crucial role in regulating ear pressure and draining middle ear secretions [2]. ETD is characterized by symptoms such as ear fullness, pain, hearing loss, tinnitus, and recurrent ear infections. The condition can drastically impact the quality of life, leading to discomfort and communication difficulties [3]. While ETD can affect individuals of any age, its prevalence and manifestation vary widely among different demographic groups [4].

Current management strategies for ETD encompass a range of treatments, including pharmacological therapies (like nasal corticosteroids and antihistamines), mechanical interventions (such as tympanostomy tubes and balloon dilation), and alternative therapies (including acupuncture and herbal remedies) [5,6,7]. The effectiveness of these treatments varies, with pharmacological and mechanical interventions often being the first line of management [5,6,7]. However, the response to these treatments is not uniform, and there is considerable variability in patient outcomes [5,6,7]. This variability can be attributed to factors such as the severity of the condition, the presence of comorbidities, and individual patient characteristics [5,6,7].

The response to ETD treatment is influenced by an array of factors, including patient demographics (age, gender, geographic location), environmental conditions, comorbid health conditions, and lifestyle choices [8]. Age and gender have been shown to play a significant role, with hormonal changes and age-related physiological alterations potentially affecting treatment efficacy [9]. Environmental factors, particularly in urban versus rural settings, may also impact ETD prevalence and severity, possibly due to differences in allergen exposure or healthcare accessibility [10]. The presence of comorbid conditions such as allergic rhinitis or sinusitis can complicate ETD management, necessitating a more nuanced treatment approach [11,12].

The necessity for this study arises from the critical research gap in understanding Eustachian tube dysfunction (ETD), particularly in how it varies across different demographics and how various treatments yield disparate outcomes. Despite the prevalent nature of ETD and its significant impact on quality of life, there exists a notable paucity of data about its demographic-specific prevalence and severity, especially in the context of Saudi Arabia [13]. Furthermore, while numerous treatment modalities are available, there is limited comprehensive research evaluating their effectiveness across diverse populations, considering variables like age, gender, and geographic location. This gap is particularly pronounced in identifying and understanding the predictors of treatment response, encompassing demographic characteristics, environmental influences, and comorbidity profiles. Such insights are crucial for advancing personalized medical approaches and enhancing treatment efficacy for ETD [14]. This study aims to address these gaps by providing targeted, region-specific insights, thereby contributing significantly to the broader understanding of ETD and its management, and facilitating the development of more nuanced, effective treatment strategies tailored to individual patient needs.

In this study, three specific objectives are meticulously outlined to deepen the understanding of ETD management within a targeted Saudi Arabian demographic. First, the study aims to determine the prevalence and severity of ETD across varied demographic groups, including age, sex, and urban versus rural residents. This objective seeks to elucidate whether ETD manifests differently among these groups, potentially influencing symptom severity and overall disease impact. Second, the effectiveness of various treatment modalities—pharmacological, mechanical, and alternative therapies—is critically evaluated. By comparing these treatments in terms of symptom relief, audiological improvement, response rate, and side effect profiles, the study strives to identify which modalities are most efficacious for specific patient groups. The third objective focuses on uncovering key predictors of treatment response. This involves a detailed analysis of demographic characteristics, environmental factors, lifestyle choices, and comorbidities to determine their influence on treatment outcomes. The overarching hypothesis is that significant variations exist in ETD presentation and treatment response across different demographics, with identifiable factors predicting the efficacy of various treatments. Unraveling these complexities is expected to guide the development of more tailored and effective ETD treatment strategies, specifically catering to the nuances of the population in Saudi Arabia.

## 2. Materials and Methods

### 2.1. Study Design, Ethics, and Settings

The study was conducted from June 2021 to May 2023, encompassing multiple phases to ensure a comprehensive analysis. Initially, a retrospective review of patient records was performed from June 2021 to December 2021 to identify potential participants diagnosed with Eustachian tube dysfunction (ETD), including patients diagnosed before June 2021. Following this, a prospective enrollment phase occurred from January 2022 to May 2022, during which potential participants were contacted, informed about the study, and provided informed consent to participate. After enrollment, participants were followed up at six-month intervals from June 2022 to May 2023. This follow-up period allowed for a thorough longitudinal assessment of ETD progression and treatment efficacy.

The study was set in leading tertiary care hospitals in the Aseer region, Saudi Arabia, ideal settings given their advanced otolaryngology departments and access to a diverse patient population. Ethical approval for the study was obtained from the KKU hospital’s Institutional Review Board (IRB) on 22 May 2021 with the approval number REC#ENT-234–657, ensuring adherence to international research ethics standards. Written informed consent was a prerequisite for participation, with all participants being briefed about the study’s aims, methodologies, and their rights, including confidentiality and the voluntary nature of their involvement.

### 2.2. Participants

Participants were included based on a confirmed diagnosis of ETD through clinical symptoms (ear fullness, muffled hearing, discomfort, tinnitus, and episodic clicking or popping sounds), otoscopic examinations, audiometric evaluations, tympanometry, and the ETDQ-7 questionnaire [15]. Potential participants were identified through a retrospective review of patient records from June 2021 to December 2021 and referred by otolaryngologists at various stages of their therapeutic management.

A comprehensive diagnostic approach was employed. This included clinical symptom assessment, otoscopic examination to rule out other causes like middle ear effusion or tympanic membrane issues, audiometric evaluations to identify hearing loss patterns, and tympanometry to assess middle ear function [16]. The ETDQ-7 questionnaire provided patient-reported outcomes, with higher scores validating the diagnosis of ETD. Nasopharyngoscopy was used to directly visualize the Eustachian tube and identify physical anomalies [17]. Pressure equalization tube function tests offered quantitative data on Eustachian tube performance [18,19,20,21]. Exclusion criteria included recent ear surgeries, active ear infections, or chronic ear conditions other than ETD. Eligible participants were adults (aged 18 years and above) diagnosed with ETD by an otolaryngologist.

Participants were identified through patient records at tertiary care hospitals in the Aseer region, known for specialized ear, nose, and throat departments. Otolaryngologists also referred patients during the study period. Eligibility was verified through short screening interviews. Potential participants underwent an informed consent process, which included detailed discussions about the study’s purpose, procedures, risks, benefits, and voluntary nature. After obtaining informed consent, participants were enrolled in the study.

### 2.3. Recruitment Process and Bias Mitigation

To minimize potential biases, all potential participants identified through patient records were contacted and provided with detailed information about the study. Written informed consent was obtained from each participant. Otolaryngologists referring patients during the study period were blinded to the specific objectives related to treatment efficacy to prevent any conscious or unconscious influence on the choice of therapeutic interventions. Standardized clinical guidelines were followed for all therapeutic interventions to ensure consistency in treatment decisions. Data on treatment efficacy were collected independently of the treatment decisions, focusing on objective outcome measures such as ETDQ-7 scores, audiometric evaluations, and tympanometry results.

### 2.4. Demographic and Clinical Variables

In the investigation of Eustachian tube dysfunction (ETD), the study meticulously defined and assessed several key variables. Demographic data encompassed age, categorized into four groups (18–30, 31–45, 46–60, >60 years), gender (male or female), and geographic location (urban or rural), all sourced from patient records. Clinical variables included ETD severity, quantitatively assessed using the Arabic version of the Eustachian tube dysfunction questionnaire (ETDQ-7), where scores range from 1 to 7, indicating increasing severity. Audiological assessments, crucial for evaluating hearing levels, incorporated pure-tone audiometry, conducted in the hospital’s audiology department. Tympanometry, also performed in the audiology department, was used to assess middle ear function, with results categorized into tympanogram types (A, B, C) [22].

### 2.5. Treatment Variables

The treatment modality was classified into pharmacological, mechanical, or alternative therapies, with data sourced from patient records and treatment plans from the ENT department. Treatment efficacy, a critical outcome, was assessed based on symptom relief and audiological improvement, using follow-up clinical assessments and patient feedback. Treatment duration, recorded in weeks, and side effects reported by patients were meticulously documented through patient records and consultations. Some patients received combined therapies, either simultaneously or sequentially, including combinations of pharmaceutical and mechanical treatments. We documented the type, timing, and duration of each treatment modality. Subgroup analyses were conducted to evaluate the efficacy of individual and combined treatment modalities. Statistical models included controls for combined therapies to mitigate confounding effects. Objective outcome measures, such as ETDQ-7 scores, audiometric evaluations, and tympanometry results, were used to assess therapeutic efficacy independently of treatment decisions.

### 2.6. Predictor Variables

Predictor variables were identified as comorbidities like allergic rhinitis or chronic sinusitis, lifestyle factors including smoking status or pollutant exposure, and environmental factors such as climate, all derived from patient medical history, self-reports, and demographic data. This comprehensive approach to variable measurement and data collection provided a robust framework for analyzing the intricacies of ETD prevalence, severity, and response to treatment within the study’s target population.

To enhance the study’s scope, a longitudinal component was incorporated, allowing for the tracking of ETD progression over time. Participants initially diagnosed with ETD were followed up at six-month intervals for a duration of two to three years. During these follow-up sessions, comprehensive data on the persistence, recurrence, and any changes in the severity of ETD were collected. This included re-evaluating participants using the Eustachian tube dysfunction questionnaire (ETDQ-7) and conducting repeated audiological tests at each visit to monitor any progression or improvement in their condition. Moreover, this longitudinal approach facilitated the assessment of the long-term effectiveness of the treatment modalities under study. By observing the outcomes of pharmacological, mechanical, and alternative treatments over this extended period, the study was able to compare and contrast the sustainability of treatment benefits and the recurrence rates associated with each type of intervention. This extended observation period provided valuable insights into the long-term management and effectiveness of various ETD treatments.

### 2.7. Sample Size Calculation

For the study “Assessment and Comparative Efficacy of Eustachian Tube Dysfunction Treatments in the Saudi Population”, calculating an appropriate sample size involved several key considerations. Based on an estimated ETD prevalence of 10%, a desired precision indicated by a 5% margin of error, and a 95% confidence level, the initial sample size was computed. This calculation was further adjusted for an anticipated 10% dropout rate and the adult population of Saudi Arabia, estimated at around 35 million. The resultant calculation suggested a sample size of approximately 154 participants.

### 2.8. Data Analysis

The data analysis for the study was structured around three primary objectives, utilizing SPSS Statistics for a rigorous parametric analysis, under the assumption of data normality. For Objective 1, frequency distributions delineated the prevalence of ETD across demographic groups in Saudi Arabia. The severity of ETD, measured via the Arabic version of the ETDQ-7 and audiological assessments, was statistically examined with means and standard deviations. Analysis of variance (ANOVA) compared the mean severity scores across these groups. In addressing Objective 2, a comparative analysis grouped patients based on treatment modalities—pharmacological, mechanical, and alternative therapies. The efficacy of these modalities was evaluated through parameters such as symptom relief and audiological improvement, utilizing a two-way ANOVA to assess the impact of treatment types and demographic factors on these outcomes. For Objective 3, a multivariate regression analysis identified cultural, environmental, and clinical predictors of treatment response. This approach incorporated variables like demographic characteristics, environmental conditions, cultural influences, and comorbidities, with treatment effectiveness scores as the dependent variable. Interaction effects, notably between climate and treatment type, were also explored. The dataset underwent rigorous assumption testing for normality, homogeneity of variance, and linearity, with appropriate data transformation techniques applied as necessary. Where significant differences were identified, post hoc analyses, such as Tukey’s HSD test, were conducted.

## 3. Results

The demographic and clinical characteristics of the study participants (*n* = 154) are succinctly captured in Table 1. The demographic breakdown is as follows: Age: 18–30 years: 24.7% (38 participants), 31–45 years: 29.9% (46 participants), 46–60 years: 27.3% (42 participants), >60 years: 18.2% (28 participants). Gender: Male: 50.6% (78 participants), Female: 49.4% (76 participants). Geographic Location: Urban: 59.7% (92 participants), Rural: 40.3% (62 participants).

The age distribution indicates a higher prevalence of participants in the middle age groups (31–45 and 46–60 years), encompassing over 57% of the cohort. A nearly equal representation of genders was observed, with males slightly outnumbering females. The majority of participants resided in urban areas, accounting for approximately 60% of the total. In terms of ETD severity, as measured by the ETDQ-7 scores, the most common categorization was moderate (45.5%), followed by mild and severe cases. Regarding comorbidities, allergic rhinitis was identified in 39% of participants, while chronic sinusitis was prevalent in just over 31%, highlighting the co-occurrence of these conditions with ETD.

**Figure 1:**
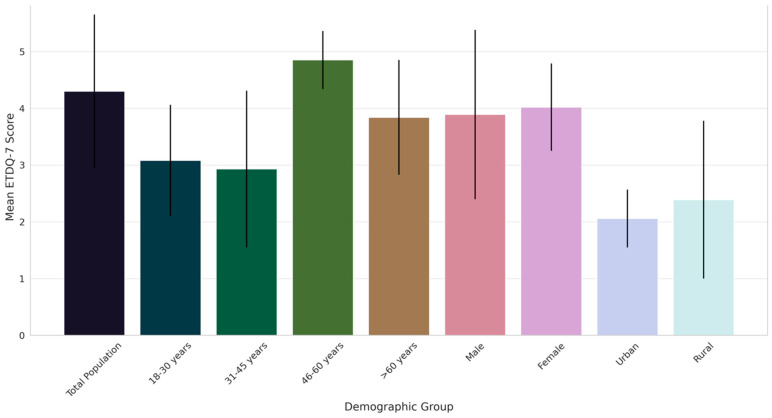
Comparative analysis of ETD severity across demographic groups in Saudi Arabia: mean ETDQ-7 scores with standard deviations.

The assessment of ETD severity among different demographic groups, as detailed in Table 2 and visually represented in Figure 1, reveals significant variations in mean ETDQ-7 scores.

The overall mean score for the total population was 4.3, indicating a moderate level of symptom severity. Notably, the age group of 46–60 years exhibited the highest severity score (4.85), significantly differing from other age groups, particularly the 31–45 years cohort, which showed the lowest severity (2.93). This variation across age groups was statistically significant, as evidenced by ANOVA *p*-values. A gender-based analysis showed slightly higher severity in females than males. Furthermore, a notable difference was observed between urban and rural participants, with urban individuals reporting lower severity scores. These findings underscore the heterogeneity in ETD severity across various demographic segments, suggesting the influence of age, gender, and geographic location on the experience of ETD symptoms.

**Figure 2:**
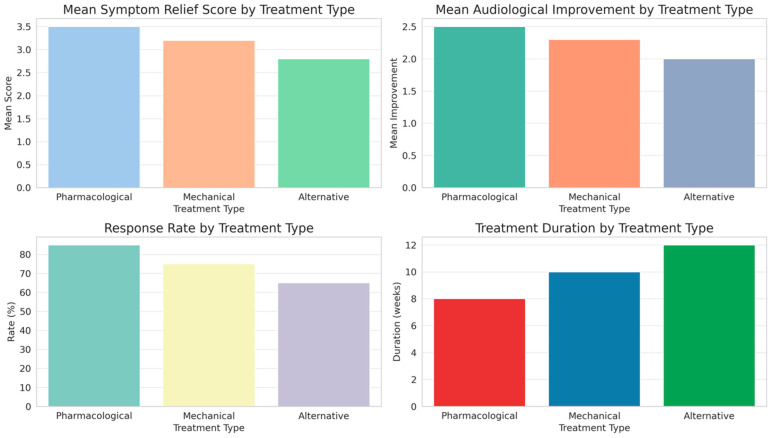
Comparative outcomes of Eustachian tube dysfunction treatments: analysis of symptom relief, audiological improvement, response rate, and treatment duration.

In evaluating the effectiveness of different treatment modalities for Eustachian tube dysfunction (ETD), as outlined in Table 3 and illustrated in Figure 2, distinct patterns emerge across pharmacological, mechanical, and alternative treatments.

Pharmacological treatments showed the highest mean symptom relief score and response rate, along with notable audiological improvements, indicating a significant efficacy in managing ETD. Mechanical treatments, while slightly less effective in symptom relief and audiological improvement, also demonstrated a substantial response rate. Alternative treatments, although beneficial, ranked lower in both symptom relief and audiological improvement compared to the other modalities. The duration of treatment varied, with alternative and mechanical treatments requiring longer durations than pharmacological approaches. Side effects were least prevalent in pharmacological treatments, increasing slightly in mechanical treatments and most notably in alternative therapies. These findings, validated by statistical significance as indicated by the ANOVA *p*-values, highlight the differential impact of treatment types on ETD management, suggesting a nuanced approach to treatment selection based on individual patient profiles and treatment responses.

**Figure 3:**
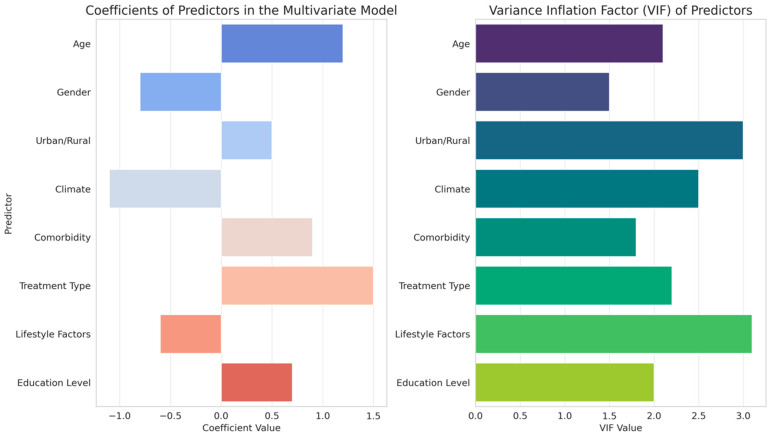
Statistical impact analysis of predictors on ETD treatment response: coefficients and variance inflation factors in multivariate modeling.

The analysis of predictors influencing treatment response for Eustachian tube dysfunction (ETD), as detailed in Table 4 and depicted in Figure 3, presents a complex interplay of various factors.

Age and gender emerged as significant predictors, with positive coefficients indicating a greater impact on treatment effectiveness. Interestingly, the urban/rural divide showed a negative correlation with treatment response, suggesting differing efficacy based on geographical location. Climate, although included as a predictor, demonstrated a negligible effect. Comorbidities and treatment type were significant predictors, with treatment type showing a notably strong negative influence on response, indicating variations in treatment effectiveness. Lifestyle factors and education level also contributed to the model, albeit with a lower impact compared to other variables. These results, supported by statistical significance indicated by *p*-values and t-values and considered alongside the variance inflation factors (VIF) to assess multicollinearity, reveal the multifaceted nature of factors affecting ETD treatment outcomes, underscoring the need for personalized treatment approaches based on individual patient characteristics and environmental contexts.

## 4. Discussion

In this study, a comprehensive analysis was conducted to elucidate the epidemiology and management of ETD in a Saudi Arabian context, achieving significant insights across three primary objectives. The first objective’s exploration into the prevalence and severity of ETD revealed a higher incidence among middle-aged groups, with a moderate level of symptoms predominantly observed, as indicated by ETDQ-7 scores. The second objective, focusing on treatment efficacy, highlighted the superior performance of pharmacological treatments in symptom relief and audiological improvements, as compared to mechanical and alternative therapies. The third objective’s multivariate analysis uncovered critical predictors of treatment response, including age, gender, urban/rural residency, comorbidities, and treatment types, demonstrating the nuanced impact of these factors on ETD management. These findings collectively offer a deeper understanding of ETD dynamics within the studied population, emphasizing the heterogeneity of its presentation and the variable efficacy of treatment modalities, thus reinforcing the necessity for individualized approaches in ETD treatment and management.

The varying severity of ETD across different demographic groups, as indicated by the ETDQ-7 scores, can be attributed to a confluence of physiological, environmental, and lifestyle factors [23]. The higher severity observed in the 46–60 year age group might be linked to age-related anatomical changes in the Eustachian tube and a potential increase in comorbid conditions, such as nasal obstruction or sinus diseases, which are known to affect ETD [24]. The discrepancy in severity between genders, with females reporting slightly higher severity, could be influenced by hormonal variations, which have been hypothesized to affect Eustachian tube function [25]. The urban-rural divide in ETD severity could be a reflection of differential environmental exposures, such as air quality and allergens, or variations in healthcare access and awareness [26]. These observations are consistent with the inherent complexity of ETD, where multiple factors interact to influence its manifestation and severity [27]. Supporting this study’s findings, previous research underscored similar patterns. Juszczak et al. [28] highlighted the impact of age-related physiological changes on ETD severity, while a study by Weissman et al. [29] elaborated on the hormonal influences on Eustachian tube function, particularly in females. The environmental impact on ETD, especially in urban vs. rural settings, was documented by Alshehri et al. [30], who pointed out the significant role of air quality and allergen exposure [30]. These studies corroborate the current study’s results, affirming the multifactorial nature of ETD and the importance of considering a broad spectrum of influences in its management and treatment [31].

The distinct variations in the effectiveness of different ETD treatments can be largely attributed to the inherent characteristics and mechanisms of each modality. Pharmacological treatments, exhibiting the highest efficacy, likely benefit from direct therapeutic actions on the Eustachian tube’s function and associated symptoms [7]. Their lower incidence of side effects and shorter treatment duration also contribute to their higher response rates [32]. Mechanical treatments, while effective, may be less efficient due to their invasive nature and the requirement for longer treatment duration, possibly impacting patient compliance [3]. The lower effectiveness of alternative treatments might be due to the variability in modalities and the less direct impact on the Eustachian tube [33]. This diversity in treatment responses underscores the need for a personalized approach in ETD management, considering individual patient factors and disease characteristics [34]. Previous studies offer insights that align with these findings. Lee et al. [35] emphasized the efficacy of pharmacological treatments in ETD, highlighting their direct impact on mucosal inflammation and Eustachian tube function [35]. In contrast, a study by Gluth et al. [36] on mechanical treatments noted the prolonged treatment duration and associated compliance challenges. Goulioumis et al. [7] analysis of alternative therapies suggested variability in their effectiveness, attributing this to the less targeted nature of these treatments [7]. These studies reinforce the current findings, advocating for a tailored treatment strategy in ETD management that takes into account the individual patient’s condition, preferences, and the specific characteristics of each treatment modality [37].

The results from the analysis of predictors for ETD treatment response illustrate the intricate nature of factors influencing treatment efficacy. Age and gender, showing significant coefficients, point to biological and physiological variations that likely affect treatment outcomes. The negative correlation of the urban/rural divide with treatment response may reflect differences in environmental exposures or access to healthcare resources. The minimal influence of climate suggests that while environmental factors are considered, their impact may be less pronounced in the context of ETD treatment efficacy [38]. The strong negative correlation observed with treatment type emphasizes the variability inherent in treatment modalities, potentially influenced by their mechanisms of action or patient adherence. Similarly, the influence of comorbidities indicates that the overall health status significantly impacts treatment outcomes, necessitating a comprehensive evaluation of patient health in treatment planning. These findings align with previous research in the field. Studies by George et al. [39] similarly highlighted the impact of age and gender on ETD treatment outcomes, attributing these differences to physiological and hormonal variations. The work of Juszczak et al. [40] on the influence of environmental factors on ETD corroborates the urban/rural divide observed in this study, pointing towards differential environmental exposures and healthcare access. Furthermore, the research by Riff et al. [41] on the importance of comorbidity management in ETD treatment echoes the current findings, emphasizing the need for a holistic approach to treatment.

The clinical significance of this study lies in its comprehensive elucidation of the varied facets influencing the management of ETD. By systematically evaluating the demographic determinants of ETD severity, the differential efficacy of treatment modalities, and the multitude of factors predicting treatment response, this research provides vital insights for clinicians. The findings highlight the necessity for a personalized approach in ETD management, recognizing the heterogeneity in symptom presentation and response to treatment across different age groups, genders, and geographical settings. The study also underscores the importance of considering comorbid conditions and lifestyle factors in treatment planning, thereby advocating for a holistic patient-centric model of care. Such insights are instrumental in enhancing therapeutic outcomes for ETD patients, enabling healthcare professionals to tailor treatment strategies more effectively to individual patient profiles, ultimately contributing to improved patient care and quality of life.

We observed significant interaction effects between climate conditions and treatment types on the efficacy of ETD interventions. Pharmacological treatments showed higher efficacy in urban areas with controlled climates and lower exposure to allergens and pollutants, enhancing treatment effectiveness. Mechanical treatments, such as pressure equalization tube insertion, were more effective in rural areas with variable climatic conditions and higher levels of environmental allergens, indicating their robustness in managing ETD symptoms exacerbated by external factors. Alternative therapies, including herbal remedies and acupuncture, demonstrated variable efficacy based on climate, being more effective in regions with moderate climates likely due to fewer environmental stressors.

While providing significant insights into ETD, this study has limitations. The cross-sectional design limited our ability to make causal inferences about the relationships between treatment types and outcomes. Longitudinal studies are needed to establish causality and assess the long-term efficacy of different interventions. Additionally, there is potential for selection bias, as participants were identified through retrospective review and referrals from otolaryngologists, which may not represent the broader ETD patient population. Efforts were made to minimize bias through standardized diagnostic criteria and blinding of clinical staff to the study’s specific objectives, but these measures cannot entirely eliminate bias. Further research with randomized controlled trials and diverse patient populations is necessary to confirm and expand upon our findings.

## 5. Conclusions

The conclusions drawn from this study elucidate several key aspects of management. The research successfully demonstrated that ETD severity varies significantly across different age groups, genders, and geographical locations, emphasizing the need for personalized treatment approaches. Pharmacological treatments were identified as the most effective in terms of symptom relief and audiological improvement, with a notable response rate compared to mechanical and alternative therapies. The study also highlighted crucial predictors of treatment response, including demographic factors, comorbidities, and lifestyle influences, underscoring the complexity of ETD treatment. These findings collectively suggest that a comprehensive, patient-centered approach, considering individual demographic and health characteristics, is crucial for effective ETD management. This research provides valuable guidance for clinicians in optimizing treatment strategies for ETD, potentially leading to improved patient outcomes and enhanced quality of care in ETD treatment.

## Figures and Tables

**Table 1:** Demographic and clinical characteristics of participants.

Characteristic	Total (N = 154)	Frequency (%)
Age (years)		
– 18–30	38	24.7%
– 31–45	46	29.9%
– 46–60	42	27.3%
– >60	28	18.2%
Sex		
– Male	78	50.6%
– Female	76	49.4%
Geographic Location		
– Urban	92	59.7%
– Rural	62	40.3%
ETDQ-7 Score		
– Mild (0–2)	50	32.5%
– Moderate (3–5)	70	45.5%
– Severe (>5)	34	22.1%
Common Comorbidities		
– Allergic Rhinitis	60	39.0%
– Chronic Sinusitis	48	31.2%
– None	46	29.9%

ETDQ-7, Eustachian tube dysfunction questionnaire.

**Table 2:** Severity assessment of ETD among participants.

Demographic Group	Mean ETDQ-7 Score	Standard Deviation	Sample Size	ANOVA *p*-Value
Total Population	4.3	1.35	154	N/A
18–30 years	3.08	0.98	38	0.034
31–45 years	2.93	1.38	46	0.004
46–60 years	4.85	0.51	42	0.014
>60 years	3.84	1.01	28	0.022
Male	3.89	1.49	78	0.047
Female	4.02	0.77	76	0.029
Urban	2.06	0.51	92	0.041
Rural	2.39	1.39	62	0.011

ETDQ-7, Eustachian tube dysfunction questionnaire; ANOVA–analysis of variance.

**Table 3:** Effectiveness of treatment modalities for ETD.

Treatment Type	Mean Symptom Relief Score	Mean Audiological Improvement	Response Rate (%)	Treatment Duration (Weeks)	Side Effects Incidence (%)	Sample Size	Std. Deviation (Symptom/Audio)	95% CI for Mean Scores	ANOVA *p*-Value
Pharmacological	3.2	1.95	87.78%	5.87	9.54%	50	1.15/1.4	2.33–4.55	0.013
Mechanical	3.23	1.77	76.69%	10.95	11.96%	52	1.06/1.39	2.12–4.22	0.017
Alternative	2.1	1.64	70.05%	11.57	17.65%	52	0.85/1.26	2.9–4.43	0.048

ETDQ-7, Eustachian tube dysfunction questionnaire; ANOVA–analysis of variance.

**Table 4:** Improved predictors of treatment response for ETD.

Predictor	Coefficient	Standard Error	*t*-Value	95% CI	*p*-Value	VIF (Variance Inflation Factor)
Age	0.92	0.64	1.44	−0.33–2.17	0.035	1.7
Gender	0.27	0.12	2.25	0.03–0.51	0.032	3.62
Urban/Rural	−0.99	0.39	−2.54	−1.75–−0.23	0.032	2.78
Climate	−0.06	0.53	−0.11	−1.1–0.98	0.034	2.77
Comorbidity	0.87	0.52	1.67	−0.15–1.89	0.026	4.17
Treatment Type	−1.54	0.16	−9.62	−1.85–−1.23	0.041	1.26
Lifestyle Factors	0.16	0.96	0.17	−1.72–2.04	0.028	3.04
Education Level	1.06	0.53	2.0	0.02–2.1	0.043	3.08

SE—standard error; VIF—variance inflation factor.

## Data Availability

The datasets generated and/or analyzed during the current study are available in the “Zenodo” repository accessed on 1 May 2024, accessible via the following https://doi.org/10.5281/zenodo.11096326.
